# Qualitative head-to-head comparison of headlamp and microscope for visualizing 5-ALA fluorescence during resection of glioblastoma

**DOI:** 10.3171/2021.10.FOCVID21181

**Published:** 2022-01-01

**Authors:** Fraser Henderson, Evgenii Belykh, Alexander D. Ramos, Theodore H. Schwartz

**Affiliations:** 1 Department of Neurological Surgery, Weill Cornell Medical School, New York, New York; and; 2 Department of Neurological Surgery, Rutgers New Jersey Medical School, Newark, New Jersey

**Keywords:** glioblastoma, fluorescence-guided surgery, 5-ALA, protoporphyrin IX

## Abstract

Fluorescence-guided surgery (FGS) for high-grade gliomas using 5-aminolevulinic acid has become a new standard of care for neurosurgeons in several countries. In this video the authors present the case of a man with glioblastoma who underwent FGS in which similar images of the operative field were acquired alternating between the microscope and a new commercially available headlight, facilitating the comparison of visualization quality between the two devices. The authors also review some of the principles of fluorescence-guidance surgery that may explain the improved brightness and contrast that they observed when using the headlamp versus the microscope.

The video can be found here: https://stream.cadmore.media/r10.3171/2021.10.FOCVID21181

## Transcript

Fluorescence-guided surgery for high-grade glioma has become a new standard of care in the United States following FDA approval of 5-aminolevulinic acid (5-ALA), trade name Gleolan.^1^ Patient ingestion of precursor molecule 5-ALA several hours before resection allows it to pass through an open blood-brain barrier, preferentially into tumor cells, where it becomes metabolized into the fluorescent drug protoporphyrin IX (PpIX). Studies have demonstrated not only safety but increases in progression-free survival in comparison to microsurgery without fluorescence.^2,3^ Though neurosurgical microscopes have incorporated filters into their systems to permit this fluorescence-guided approach, improved visualization techniques remain a paramount interest for glioma surgeons.^4^ One solution has been to develop a wearable fluorescence-guided headlamp for excitation of the fluorophore in association with absorption filters built into surgical loupes to remove extraneous or distracting wavelengths.

In this video we present a man with glioblastoma who underwent fluorescence-guided surgery in which similar images of the operative field were acquired alternating between the microscope and a new commercially available headlight, facilitating the comparison of visualization quality between the two devices. We also review some of the principles of fluorescence-guidance surgery that may explain the differences we observed between the microscope and the headlamp.

### 1:42 Brief Review of Fluorescence Guidance Principles.

To review, both illumination devices create a uniform beam of light with wavelength of about 405 nm. Four hundred five–nanometer light corresponds to the peak absorption spectrum of PpIX, allowing for its excitation and emission of lower-energy photons at about 630 nm. Four hundred five–nanometer light that does not strike PpIX reflects from the tissue. This results in two spectral beams of light: reflected blue from nontumor and newly formed fluorescent red from tumor returning through the optical filter of the microscope or loupes that attenuate intensity of the violet-blue light but permit the passage of the red light.

Since both 405-nm light and 630-nm light are within the dynamic range sensitivity of a human eye, we can perceive the image as shades of blue and red, thereby enabling a visual distinction between neoplastic and nonneoplastic tissue. While the positive predictive value for 5-ALA fluorescence has approached 100% in most high-grade glioma series, its negative predictive value (NPV) in surgeries using the microscope filter has ranged from 22% to 69% in a recent review,^5^ a finding partly explained by the extensively infiltrating nature of high-grade gliomas and partly from the lack of blood-brain barrier breakdown in peripheral regions with lower-density tumor infiltration.

### 3:01 Patient Presentation.

In this case, an 85-year-old left-handed Russian-speaking man presented with worsening word-finding difficulty. Examination revealed dysnomia and dyscalculia. MRI of the brain revealed a heterogeneously enhancing mass in the left temporal lobe with an adjacent satellite nodule, likely glioblastoma. Neuropsychological testing confirmed candidacy for awake language mapping.

### 3:25 Operative Plan.

The patient was administered 5-ALA oral solution 20 mg/kg [Gleolan, NXDC] 3 hours prior to surgery. He was taken to the operating theater. Under local anesthesia with conscious sedation, a head clamp was applied and secured to the bed where navigation was registered. A wide craniotomy was performed over the tumor. The patient was awakened and language mapping was performed in both English and Russian to outline a safe corticotomy. No essential language areas were found overlying the expected incision.

### 3:56 Wearable Fluorescence-Guided System.

Fluorescence-guided resection was performed alternating between the microscope and headlamp. Headlamp images were created using a high-definition LED headlight (TriBeam HDiTM Headlight firmware version 1.0, Powerpack firmware version 2.0, Designs for Vision) emitting 405-nm and 450-nm wavelength light combined with loupes containing filters that block light beneath 430 nm [REVEAL, Designs for Vision]. The loupes contain two filters in each telescope and one in the carrier lens that provide emission filtering in both the magnification and eyeglass lens optics. The headlamp system features a wireless Bluetooth foot pedal pairing function to alternate between natural light and filtered light. Headlamp images were recorded using an iPhone 12 Pro camera [Apple Inc.] paired with a smartphone filter adapter [Designs for Vision]. Microscope images were acquired using the built-in system [Zeiss KINEVO].

### 4:47 Comparison of Microscope and Headlamp.

Theater lights were turned down during fluorescence visualization. Microscope images were recorded directly through the microscope. The conventional microscope (Zeiss KINEVO) was activated into BLUE400 fluorescence mode for PpIX visualization. As demonstrated in the video, the PpIX fluorescence provided by the headlamp is dramatically brighter than that provided by the microscope. Indeed, the headlamp visualization provided a sharper contrast between tumor and normal brain, in both the resection cavity and ex vivo on the specimen table. The headlamp performed especially well when the cavity contained blood, since hemoglobin absorbs much of the light in the fluorescent emission spectrum, thereby obfuscating fluorescence detection.

Three groups of factors affect the intensity of PpIX fluorescence.^6^ First, those related to the excitation light, such as 1) light power, 2) distance to the tissue, 3) excitation light spectrum, and 4) light beam shape. Second, those related to the fluorophore in the tissue, including 1) timing of surgery within the optimal window of maximal PpIX accumulation, 2) blood products obscuring the excitation light, and 3) degradation of fluorescence over time due to photobleaching. The third category are factors related to the detection device, such as 1) distance from the tissue to the detector, 2) sensitivity of the detector, 3) exposure time, 4) spectrum width that is being detected, and 5) any splitting of the emitted light into different pathways by the hardware.

This headlamp employs a powerful excitation source that allows for robust PpIX excitation at the optimal spectral range. “Light power density” depends on the working distance between source and target, as presented in this graph. Assuming the shape of the headlamp illumination light beam is similar to the Zeiss microscope, the curve presented on the graph can be approximated to the headlamp. The graph, based on previously reported data for the Zeiss microscope,^4^ equates a headlamp positioned 43 cm (17 inches) from target [data provided by Designs for Vision] with about same light power density as a microscope in BLUE400 mode positioned at 25 cm (10 inches) distance (~ 6.5 mW/cm^2^). By attaching to the surgeon’s forehead, the source of the excitation moves with the gaze of the surgeon more quickly than the microscope, which must be moved separately. Moreover, rather than employing a hand switch, as used by the microscope, the headlamp utilizes a wireless pedal that allows for rapid alternation between white light and two different settings of blue light to optimize visualization of the surrounding brain so the surgeon can work under blue light. Using pure 405-nm excitation, the operator would mostly see red PpIX fluorescence on a background that is close to normal colors. The second mode, blending 405-nm and 450-nm excitation lights together, includes red fluorescent light but also surrounding tissue without PpIX that appears bluish because of reflected 450-nm light, thereby enhancing contrast between tissue types.

Here we show a diagram of the power of the excitation blue light measured at various distances from the microscope^4^ (Graph 1). Greater excitation power creates brighter fluorescence. Therefore, a headlight used at 17 inches from the tissue (labeled as a star) has greater blue light power than a surgical microscope positioned at the same distance, and comparable excitation power to when the microscope is used at the usual distance from the tissue. Additionally, TriBeam HDiTM Headlight uses a longpass filter that allows 91% light transmission efficiency which is devoid of the complex microscope optical system, including beam splitter, which inevitably reduces efficiency of eventual light transmission to the surgeon’s eye or microscope camera.^7^ Surgical loupes attached to the headlamp are manufactured with either ×2.5 or ×3.5 magnification, versus the Zeiss microscope, which achieves up to ×10–×12 magnification at 30 cm.

### 8:41 Limitations.

One limitation to this comparative study is that digital recordings from different systems that may misrepresent what the surgeon’s eye is receiving through the microscope oculars. Light entering the microscope splits on its way to the camera, decreasing brightness relative to the surgeon’s actual view. We present a better approximation of the surgeon’s oculars’ view by increasing the still-image brightness by 60%. Unfortunately, a tumor/brain ratio (TBR) comparison cannot be performed in this study because data are not within the dynamic range due to the microscope images being underexposed for red light. REVEAL images in this study showed a TBR of 4.

Further study of headlamp-assisted fluorescence-guided surgery will determine whether this technology can improve the negative predictive value of 5-ALA and assure that there are not excessive false positives. Such a study will require analysis of histological specimens.

### 9:34 Surgical Outcome.

The patient recovered in a light-regulated environment for 48 hours according to institutional protocol. Postoperative MRI confirmed gross-total resection, including the satellite nodule. The patient was monitored after an episode of confusion on postoperative day 2, but this resolved and EEG showed no seizures. He was discharged to home 5 days after surgery and seen in follow-up with no complications and referred to neuro-oncology to begin his adjuvant care.

## Disclosures

Dr. Schwartz reports being a consultant for RPW Technology, Integra, Elliquence, and NX Development; and owning stock in RPW Technology, MIVI Neuroscience, Serenity Medical Inc., Neurotechnology Investors LLC, and EndoStream Medical, all of which is outside the submitted work.

## Author Contributions

Primary surgeon: Schwartz. Assistant surgeon: Ramos. Editing and drafting the video and abstract: Schwartz, Henderson, Belykh. Critically revising the work: Schwartz, Henderson, Belykh. Reviewed submitted version of the work: all authors. Approved the final version of the work on behalf of all authors: Schwartz. Supervision: Schwartz.
